# AAU-Specific RNA Cleavage Mediated by MazF Toxin Endoribonuclease Conserved in *Nitrosomonas europaea*

**DOI:** 10.3390/toxins8060174

**Published:** 2016-06-04

**Authors:** Tatsuki Miyamoto, Akiko Yokota, Satoshi Tsuneda, Naohiro Noda

**Affiliations:** 1 Department of Life Science and Medical Bioscience, Waseda University, Shinjuku-ku, Tokyo 162-8480, Japan; tatuki-miyamoto@asagi.waseda.jp; 2 Biomedical Research Institute, National Institute of Advanced Industrial Science and Technology (AIST), Tsukuba, Ibaraki 305-8566, Japan; akiko-yokota@aist.go.jp

**Keywords:** *Nitrosomonas europaea*, toxin-antitoxin system, MazF, sequence-specific endoribonuclease

## Abstract

*Nitrosomonas europaea* carries numerous toxin-antitoxin systems. However, despite the abundant representation in its chromosome, studies have not surveyed the underlying molecular functions in detail, and their biological roles remain enigmatic. In the present study, we found that a chromosomally-encoded MazF family member, predicted at the locus NE1181, is a functional toxin endoribonuclease, and constitutes a toxin-antitoxin system, together with its cognate antitoxin, MazE. Massive parallel sequencing provided strong evidence that this toxin endoribonuclease exhibits RNA cleavage activity, primarily against the AAU triplet. This sequence-specificity was supported by the results of fluorometric assays. Our results indicate that *N. europaea* alters the translation profile and regulates its growth using the MazF family of endoribonuclease under certain stressful conditions.

## 1. Introduction

*Nitrosomonas europaea* is a chemolithoautotrophic bacterium in the beta-subdivision of Proteobacteria [1]. It inhabits aquatic and terrestrial environments and acquires reductants by oxidizing ammonia to nitrite [2]. Since ammonia oxidization is a key reaction in both the ecological nitrogen cycle and environmental engineering [3], the physiological responses of *N. europaea* in a variety of environments have been investigated; it is now widely accepted that this bacterium responds sensitively to environmental changes such as temperature, ammonia concentration, pH, nitrite concentration, inorganic substances, and organic compounds [4,5,6,7,8].

The genome information of this bacterium was previously published [9]. Interestingly, *N. europaea* bears a large number of putative toxin-antitoxin (TA) systems, which are stress-responsible genetic modules widespread in bacterial and archaeal lineages. This indicates that *N. europaea* acclimates to variable environments using these systems [10]. However, no studies have surveyed the molecular functions of these TA systems conserved in this chemolithoautotroph.

TA systems are typically encoded by a set of two genes: one for a long-lived toxin that inhibits vital processes in microbial cells and the other for a short-lived antitoxin that neutralizes toxin activity [11]. In most cases, antitoxins are preferentially degraded under stressful conditions. This results in toxin liberation and subsequent growth arrest [11]. Although the cellular targets of toxins are diverse, most previously identified toxins are known to arrest microbial growth by cleaving intracellular RNAs [12].

*Escherichia coli* MazF, which belongs to the MazEF family with its cognate antitoxin MazE, is one of the best-characterized toxins [13]. Under stressful environments, MazF specifically cleaves cellular RNAs at ACA sites irrespective of the ribosome [14], serving as a post-transcriptional regulator [15,16]. Interestingly, MazF homologues are well-conserved in the prokaryotic domain [10,17]. Additionally, they cleave discrete RNA sites based on recognition length and sequences [14,18,19,20,21,22,23,24,25,26,27,28]. Therefore, MazF homologues are thought to play diverse biological roles; indeed, they have been implicated in programmed cell death [29], dormancy [30], phage abortive infection [31,32], and pathogenicity [21,22,33].

In the present study, we showed a MazF homologue, predicted at NE1181 in the *N. europaea* chromosome (MazF_NE1181_), is a toxin endoribonuclease, which forms a TA pair together with its cognate antitoxin MazE, encoded by the NE1182 locus (MazE_NE1182_). Using a combination of RNA-seq and fluorometric assays, this enzyme was found to cleave AAU sites in a sequence-specific manner. These results indicate that *N. europaea* translation is altered by the action of this enzyme during specific environmental stresses.

## 2. Results

### 2.1. Enzymatic Activity of MazF_NE1181_

**Figure 1:**
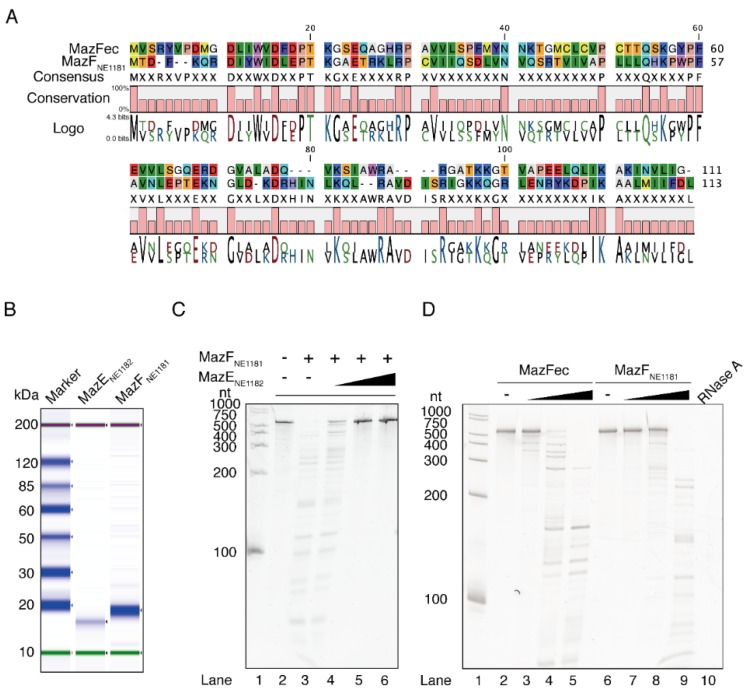
A MazF homologue isolated from *N. europaea*. (**A**) Pairwise alignment of two MazF sequences; (**B**) molecular weight and purity of MazEF pair; (**C**) enzymatic activity of MazE_NE1182_ and MazF_NE1181_. Lane 1, ladder; lane 2, control reaction without enzymes; lanes 3–6, 30 pmol of MazF_NE1181_ was added. For lanes 4–6, 20, 60, and 180 pmol of MazE_NE1182_ was added, respectively; and (**D**) cleavage pattern of MazFec and MazF_NE1181_; Lane 1, ladder; lanes 2 and 6, control reactions without any enzymes; lanes 3–5, 10, 50, and 250 ng of MazFec was added, respectively; lanes 7–9, 10, 50, and 250 ng of MazF_NE1181_ was added, respectively; lane 10, 100 ng of RNase A was added as a control.

MazF_NE1181_ codes for a 113-residue protein and shows 26.9% identity to *E. coli* MazF (MazFec) (Figure 1A), but its function remains unclear. We purified histidine-tagged MazF_NE1181_ (Figure 1B) and then examined its endoribonuclease activity. We incubated a 533-nt RNA with this enzyme, and observed RNA degradation (Figure 1C, Lane 3), which suggests that MazF_NE1181_ is a toxin endoribonuclease. Next, in order to rule out the potential contamination with RNases, a cognate MazE antitoxin (MazE_NE1182_) was purified (Figure 1B). The addition of MazE_NE1182_ was shown to block RNA cleavage (Figure 1C, Lanes 4–6), demonstrating that it is specifically mediated by MazF_NE1181_. Moreover, the cleavage patterns differed between MazFec- and MazF_NE1181_-treated RNAs (Figure 1D). Taken together with the fact that both MazF proteins yielded numerous RNA fragments, it appears that MazF_NE1181_ recognizes short unique sequences.

### 2.2. Cleavage Sequence Identification Using Massive Parallel Sequencing

**Figure 2:**
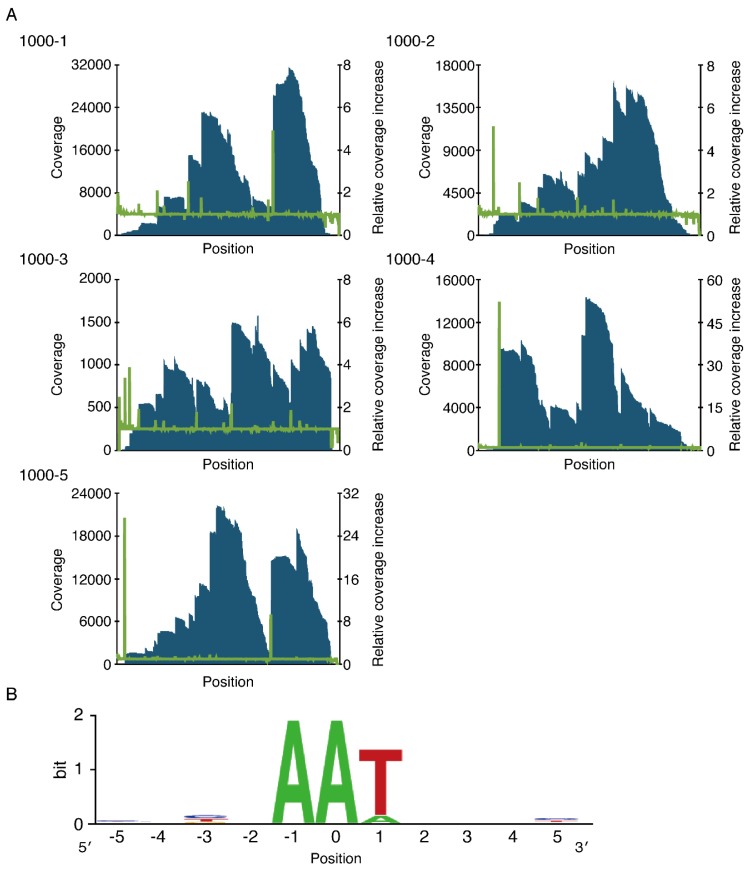
Analysis of the cleavage sequence of MazF_NE1181_. (**A**) Graph of the coverage (blue bar) and relative coverage increase (green line); and (**B**) conserved sequence around nucleotide positions with increased coverage. Nucleotide position with significant increases in coverage was set to zero.

We recently developed an RNA-seq-based approach for cleavage sequence determination [25]. Hence, we attempted to define the cleavage specificity of MazF_NE1181_ using this approach (see Appendix A). When analyzing the MazF_NE1181_-cleaved RNA sites, we found that the AAT triplet was highly conserved (Figure 2B), suggesting that MazF_NE1181_ preferably recognizes and cuts RNAs at the unique triplet AAU. Furthermore, since the coverage significantly increased at the second A-residue (Table S1 and Figure 2B), MazF_NE1181_ likely cleaves RNAs between the first and second adenines.

### 2.3. Cleavage-Specificity Validation Based on Fluorescence Resonance Energy Transfer

To further confirm its sequence-specificity, we next examined whether MazF_NE1181_ cleaves fluorescent-modified oligonucleotides (Table 1) based on fluorescence resonance energy transfer (see Appendix B) [25,34].

**Figure 3:**
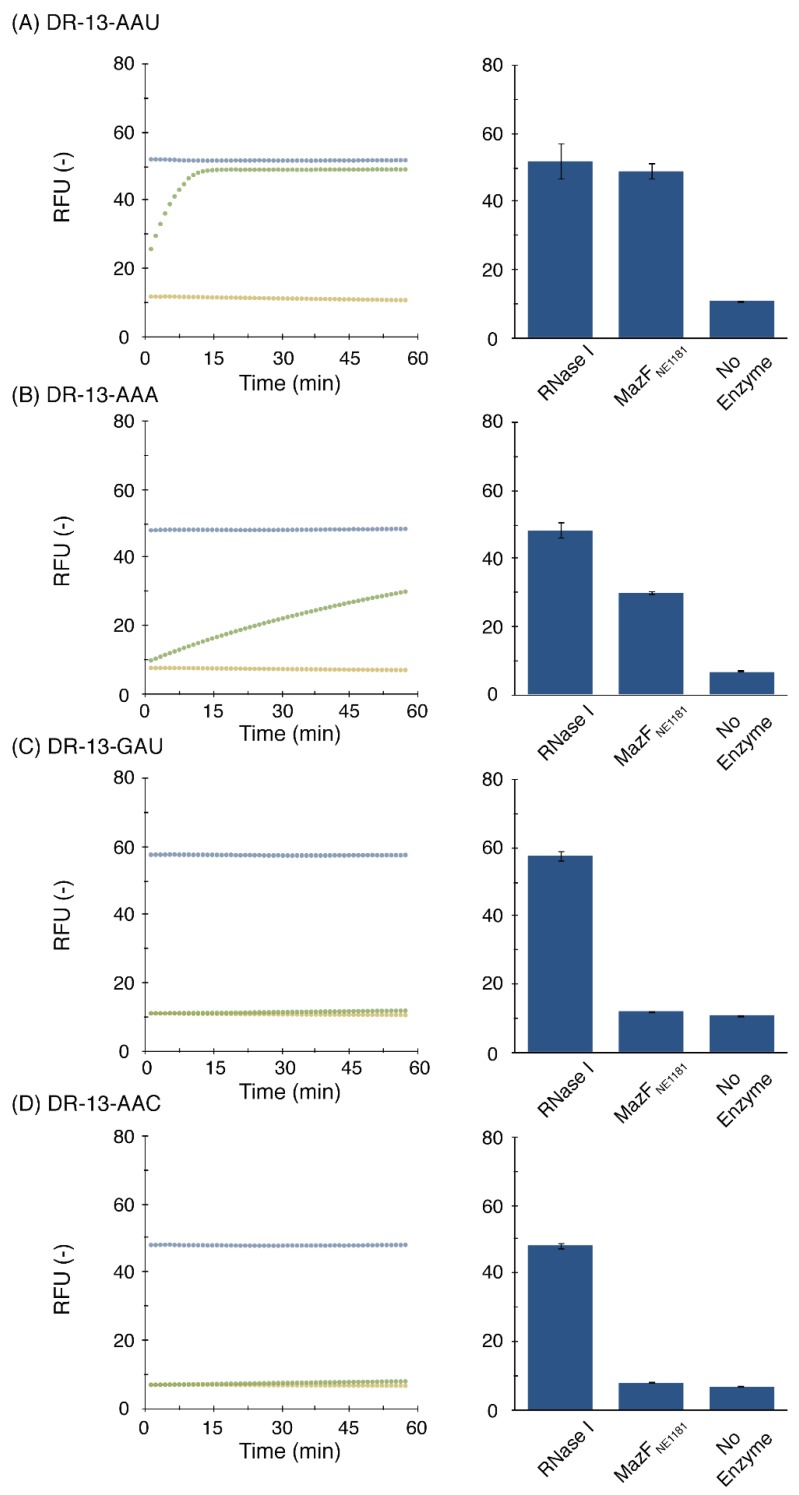
MazF_NE1181_-mediated sequence-specific RNA cleavage. Two hundred nanograms of MazF_NE1181_ (green) was incubated with 20 pmol of fluorescent-modified oligonucleotides; (**A**) DR 13-AAU; (**B**) DR-13-AAA; (**C**) DR-13-GAU; and (**D**) DR-13-AAC. In the control reactions, fluorescent intensities in the presence of 1 U of RNase I (blue) and in the absence of enzymes (yellow) at each time point (left) and end point (right) were measured.

In agreement with the results obtained from RNA-seq, when a chimeric DNA/RNA oligonucleotide DR-13-AAU was first treated with MazF_NE1181_, fluorescent intensity rapidly increased (Figure 3A), verifying that AAU is the target of this enzyme. Therefore, we next examined whether AAA was also susceptible to this enzyme, since this triplet was detected by massive parallel sequencing (Figure 2B, Table S1). We synthesized DR-13-AAA and incubated it with MazF_NE1181_. As expected, the AAA triplet was also cleaved, but the cleavage activity was greatly weakened (Figure 3B); indeed, while DR-13-AAU was completely cleaved within 15 min, nearly 50% of DR-13-AAA remained intact, even at the end of the reaction. Thus, AAU was considered to be the main target of the enzyme.

Notably, a DNA oligonucleotide that is composed of a DNA adenine repeat (D-13-AAA) was tolerant to MazF_NE1181_ (Figure S1A). Furthermore, MazF_NE1181_-mediated RNA cleavage was nearly completely blocked for three RNA oligonucleotides (R-13-GUUGU, R-13-UCUCG, and R-13-UGACA) (Figures S1B–D), the sequences of which were derived from substrate RNA used in the RNA-seq but did not include the AAU and AAA sequences. Taking these results together with the results showing that DR-13-AAU cleavage was counteracted by the addition of MazE_NE1182_ in a dose-dependent manner (Figure S2), the possibility of contamination by DNases and RNases was, again, excluded.

Finally, we prepared two additional fluorogenic oligonucleotides (DR-13-GAU and DR-13-AAC) to investigate whether MazF_NE1181_ strictly recognizes specific sequences. As anticipated, neither DR-13-GAU, an oligonucleotide whose first RNA base A is substituted with another purine base G, nor DR-13-AAC, whose last RNA base U is substituted with another pyrimidine base C, were cleaved, demonstrating the strictness of MazF_NE1181_-recognition (Figure 3C,D). Thus, MazF_NE1181_ is a canonical toxin endoribonuclease that mainly targets the AAU sequence.

## 3. Discussion

TA systems are ubiquitous elements encoded in prokaryotic plasmids and chromosomes [10,35,36,37] and are involved in stress adaptation by modulating bacterial and archaeal growth. Although toxin molecules regulate microbial growth through a variety of mechanisms, an enormous number of toxins are known to function as RNA endoribonucleases. Based on the mode of action, these toxin endoribonucleases are classified into two categories: (i) ribosome-dependent endoribonucleases (*i.e.*, RelE, YafQ, and HigB) [38,39,40] and (ii) ribosome-independent endoribonucleases (*i.e.*, MazF, HicA, and VapC) [14,41,42].

In the current study, we demonstrated that the AAU site is the prime target of *N. europara* MazF (MazF_NE1181_) (Figure 2 and Figure 3). The consensus sequence for the MazF_NE1181_ was previously known as 5′-GAAU-3′ and 5′-AAAU-3′ [43]. However, these cleavage sequences were roughly estimated based on gel electrophoresis results. Using a combination of massive parallel sequencing and fluorometric assays, we refined the cleavage-specificity of MazF_NE1181_.

It has been well-established that transcripts without recognition sequences are tolerant to toxin endoribonucleases [21,22,44]. Accordingly, we extracted protein-coding sequences without any AAU triplets. We found that eight out of 2462 sequences were devoid of this triplet (Table 2).

Interestingly, three of eight genes were identified within *mer* operons (Table 2), which are composed of *merTPCADE* and *merR* (Table S2) [6,7]. Previously, these genes were inferred to be helpful for improving the resistance to mercury [6]. Furthermore, they were implicated in the resistance of *N. europaea* to other heavy metal stresses; in fact, these genes were significantly upregulated after heavy metal exposure [6,7]. Considering that some toxin endoribonucleases regulate gene expression by differentially destabilizing mRNAs, including recognition sequences [45], MazF_NE1181_ may function as a post-transcriptional regulator and improve heavy metal resistance by enriching the transcripts within this operon; indeed, *mazEF* expression at this locus was reported to be upregulated under zinc stress [7].

Additionally, the gene sequence in the locus NE1224 did not include AAU triplets (Table 2). RASTA-Bacteria, an automated web-based tool for identifying prokaryotic toxin-antitoxin systems [46], predicted that this gene codes for an antitoxin that comprises a TA system along with a VapC family of toxin endoribonucleases (NE1225) [35,46]. Given that protein antitoxins typically suppress the expression of the TA system by binding its promoter [47], this putative antitoxin (NE1224) may repress the expression of VapC toxin endoribonuclease, in which case *N. europaea* may utilize these endoribonucleases depending on their surroundings and acclimate to the environments by using RNAs that evade MazF_NE1181_ or VapC-catalyzed cleavage.

In conclusion, we found that MazF_NE1181_ is a functional enzyme and possesses endoribonuclease activity. In addition, this MazF homologue mainly recognizes and cleaves RNAs at AAU sites in a ribosome-independent manner. This indicates that *N. europaea* alters its translation and copes with certain stresses with the aid of this enzyme.

## 4. Materials and Methods

### 4.1. Plasmids and Oligonucleotides

The pET21c expression vector was purchased from Takara Bio Service (Shiga, Japan). pET19b expression vector encoding *mazE*_NE1182_, with the codon usage optimized for recombinant protein expression in *E. coli*, was purchased from GenScript Japan (Tokyo, Japan). pMK-T encoding *mazF*_NE1181_, whose codon usage was optimized for recombinant protein expression in *E. coli*, was purchased from Life Technologies Japan Ltd. (Tokyo, Japan). Fluorescent-modified oligonucleotides were purchased from Japan Bio Services (Saitama, Japan).

### 4.2. Plasmid Construction

pMK-T encoding *mazF*_NE1181_ and pET21c were digested with *Xho*I and *Bam*HI (Toyobo, Osaka, Japan). These linearized DNA fragments were cleaned using a MinElute PCR purification kit (Qiagen, Hilden, Germany). The *mazF*_NE1181_ fragment was then cloned into the multiple cloning sites of pET21c using a DNA ligation kit (Takara), generating the plasmid pET21c-*mazF*_NE1181_. *E. coli* strain DH5α (Nippon Gene, Tokyo, Japan) was transformed with pET21c-*mazF*_NE1181_, and this transformant was grown at 37 °C on LB plate containing 100 μg/mL ampicillin. pET21c-*mazF*_NE1181_ was extracted using the QIAprep Spin Miniprep Kit (Qiagen), and the sequence was confirmed using an AB 3500 Genetic Analyzer (Applied Biosystems, Foster City, CA, USA) according to the manufacturer’s protocol.

### 4.3. Expression of MazE_NE1182_

*E. coli* strain BL21 (DE3) cells (BioDynamics Laboratory Inc., Tokyo, Japan) were transformed using pET19b-*maz*E_NE1182_. These cells were pre-cultivated overnight in LB medium supplemented with 100 μg/mL ampicillin at 37 °C. Afterward, they were inoculated into 1 L of LB medium containing 100 μg/mL ampicillin. MazE_NE1182_ was induced by the addition of 1 mM isopropyl β-d-1-thiogalactopyranoside, when OD600 reached approximately 1.0. After 3.5 h of incubation, the cells were harvested by centrifugation at 7000 *g*, and stored at −80 °C until further use.

### 4.4. Purification of MazE_NE1182_

Recombinant MazE_NE1182_ was purified as described previously with minor modifications [25]. *E. coli* cells containing MazE_NE1182_ were thawed on ice and resuspended in 14 mL of binding buffer (20 mM sodium phosphate (pH 8.0), 300 mM NaCl, 40 mM imidazole, and 5 mM 2-mercaptoethanol). Afterward, these cells were incubated on ice for 5 min in the presence of 0.2 mg/mL lysozyme. The cells were lysed by sonication and collected by centrifugation at 7000 *g* for 15 min. Afterward, the supernatant was filtered through a 0.45-μm membrane (Millex, Darmstadt, Germany). After equilibrating a 1-mL His-Trap FF column (GE Healthcare, Little Chalfont, UK), the supernatant was applied to the column and washed with 32 column volumes of binding buffer using AKTA pure 25 (GE Healthcare). Deca-histidine tagged MazE_NE1182_ was selectively eluted with the elution buffer using following program: flow rate, 1 mL/min; linear elution gradient, 20 column volumes; fraction size, 0.5 mL. The following composition of the elution buffer was used: 20 mM sodium phosphate (pH 8.0), 300 mM NaCl, 500 mM imidazole, and 5 mM 2-mercaptoethanol. The 38th fraction from the beginning of the elution program was used for further experiments. The molecular weight and purity were confirmed using the Agilent 2200 TapeStation P200 ScreenTape Assay (Agilent Technologies, Santa Clara, CA, USA). Protein concentration was determined using the Qubit Protein Assay Kit (Life Technologies, Carlsbad CA, USA).

### 4.5. Expression of MazF_NE1181_

*E. coli* strain BL21 (DE3) (Nippon Gene) was transformed with pET21c-*mazF*_NE1181_ via heat shock, and this transformant was pre-cultivated overnight in LB medium supplemented with 100 μg/mL ampicillin at 37 °C. Pre-cultivated *E. coli* cells were then inoculated into 1 L LB medium containing 100 μg/mL ampicillin and 3% NaCl and then incubated overnight. MazF_NE1181_ was induced by the addition of 1 mM isopropyl β-d-1-thiogalactopyranoside. After 3.5 h of incubation, the cells were harvested by centrifugation at 7000 *g* and then stored at −80 °C until use.

### 4.6. Purification of MazF_NE1181_

Recombinant MazF_NE1181_ was purified as described previously with minor modifications [25]. *E. coli* cells containing MazF_NE1181_ were thawed on ice and resuspended in 15 mL of binding buffer (20 mM sodium phosphate (pH 8.0), 0.05% Triton X-100, 300 mM NaCl, 40 mM imidazole, and 5 mM 2-mercaptoethanol). Suspended cells were then incubated on ice for 5 min in the presence of 0.2 mg/mL lysozyme. The cells were lysed by sonication and collected by centrifuging at 7000 *g* for 15 min. The supernatant was then filtered through a 0.45-μm membrane (Millex). After equilibrating a 1-mL His-Trap FF crude column (GE Healthcare), the supernatant was applied to the column and washed with 32 column volumes of binding buffer using AKTA pure 25 (GE Healthcare). Hexa-histidine tagged MazF_NE1181_ was selectively eluted, using the elution buffer, with following program: flow rate, 1 mL/min; linear elution gradient, 20 column volumes; fraction size, 0.5 mL. The elution buffer contained 20 mM sodium phosphate (pH 8.0), 0.05% Triton X-100, 300 mM NaCl, 500 mM imidazole, and 5 mM 2-mercaptoethanol. The 22th fraction from the beginning of the elution program was used for further experiments. The molecular weight and purity were confirmed using the Agilent 2200 TapeStation P200 ScreenTape Assay (Agilent Technologies). Protein concentration was determined using the Qubit Protein Assay Kit (Life Technologies).

### 4.7. Enzymatic Activity of MazF_NE1181_ and MazE_NE1182_

Synthetic RNA constructs were prepared as described in our previous study [25]. Thirty picomoles of MazF_NE1181_ were pre-incubated with 20, 60, or 180 pmol of MazE_NE1182_ at room temperature for 10 min. Following this, 100 ng of RNA 500-2 was added and the mixture was incubated at 37 °C for 30 min in MazF reaction buffer (20 mM Tris-HCl (pH 8.0), 1 mM dithiothreitol, 0.01% Triton X-100, and 4 U of recombinant RNase inhibitor (Takara)) in a final volume of 50 μL. Samples were purified by RNA Clean and Concentrator™-5 (Zymo Research, Orange, CA, USA) and the gel loading buffer II (Ambion, Austin, TX, USA) was added to each sample. They were incubated at 95 °C for 5 min and separated on a 10% polyacrylamide gel containing 7 M urea. RNA was stained using SYBR Gold (Life Technologies) and detected using a Typhoon 9210 imager (GE Healthcare).

### 4.8. Endoribonuclease Activity of MazF_NE1181_

Synthetic RNA constructs were prepared as described in our previous study [25]. RNA 500-2 was incubated with 10, 50, or 250 ng of MazFec or MazF_NE1181_ at 37 °C for 30 min in MazF reaction buffer in 25-μL reaction volume. Gel loading buffer II (Ambion) was added to each sample. These samples were incubated at 95 °C for 5 min and then separated on a 10% polyacrylamide gel containing 7 M urea. RNA was stained using SYBR Gold (Life Technologies) and then detected using a Typhoon 9210 imager (GE Healthcare).

### 4.9. Cleavage Sequence Identification

The cleavage sequence was identified using the protocols described in our previous study [25]. First, 1.5 μg of five RNA mixtures were incubated with 400 ng of MazF_NE1181_ at 37 °C for 30 min in MazF reaction buffer. Phosphorylation, barcode ligation, and sequencing library construction were performed as described by Miyamoto *et al.* [25]. Sequencing was performed using the MiSeq platform with the MiSeq 500 cycles reagent kit v2 (Illumina, San Diego, CA, USA) according to the manufacturer’s protocol. Sequence data was analyzed using CLC Genomics 7.5.1. The parameters described by Miyamoto *et al.* [25] were used for the analysis, and 25 sequences were analyzed using WebLogo [48]. The deep sequencing dataset was deposited into the DDBJ Sequence Read Archive (DRA004562).

### 4.10. Fluorometric Detection of MazF_NE1181_ Activity

The flurometric assay was performed as described previously [25]. Two hundred nanograms of MazF_NE1181_ or 1 U of RNase I (Epicentre Biotechnologies, Madison, WI, USA) were incubated with 20 pmol of fluorescent-labeled oligonucleotides in MazF reaction buffer in a total volume of 20 μL. All reactions were conducted at 37 °C in triplicate and fluorescent intensity was recorded every 1 min using a Light Cycler 480 system (Roche, Basel, Switzerland) with 483 nm excitation and 533 nm detection filters.

### 4.11. Neutralization of MazF_NE1181_-mediated Cleavage

Ten picomoles of MazF_NE1181_ were pre-incubated with 2, 10, or 50 pmol of MazE_NE1182_ at room temperature for 10 min. Afterward, 20 pmol of fluorescent-labeled oligonucleotide (DR-13-AAU) was added, and the mixture was incubated at 37 °C in MazF reaction buffer in a final volume of 20 μL. All reactions were conducted at 37 °C in triplicate and fluorescent intensity was recorded every 1 min using a Light Cycler 480 system (Roche) with 483 nm excitation and 533 nm detection filters. In the control reactions, fluorescent intensities in the presence of 1 U of RNase I (Epicentre) and in the absence of enzymes were measured.

### 4.12. Accession Numbers

The GenBank accession numbers are as follows: *mazE_NE1182_* protein sequence (WP_011111771), *mazF_NE1181_* protein sequence (WP_011111770) and artificially designed RNAs; 500-2 (AB610940), 1000-1 (AB610944), 1000-2 (AB610945), 1000-3 (AB610946), 1000-4 (AB610947), and 1000-5 (AB610948).

## Figures and Tables

**Table 1:** Fluorescent-modified oligonucleotides used in fluorometric assay.

Name	Sequence (5′ to 3′) ^a^
DR-13-AAU	AAAAAAAUAAAAA
DR-13-AAA	AAAAAAAAAAAAA
D-13-AAA	AAAAAAAAAAAAA
R-13-GUUGU	GUUGUCAUGCCGG
R-13-UCUCG	UCUCGGUGCGUUG
R-13-UGACA	UGACACGAACCGC
DR-13-GAU	AAAAAGAUAAAAA
DR-13-AAC	AAAAAAACAAAAA

^a^ Underlined letters represent RNA nucleotides and the other letters represent DNA nucleotides.

**Table 2:** Protein coding sequences without AAU sequences.

Locus	Gene Symbol	Length (bp)	Product Name
NE0390	*rpmH*	135	LSU Ribosomal protein L34
NE2575	*merE*	237	mercury resistance protein
NE0841	*merP*	276	mercury scavenger protein
NE0842	*merT*	351	mercuric transport protein
NE1224	-	264	hypothetical protein
NE1344	-	279	hypothetical protein
NE2523	-	231	hypothetical protein
NE2538	-	912	hypothetical protein

## Supplementary Materials

The following are available online at www.mdpi.com/2072-6651/8/6/174/s1, Figure S1: MazF_NE1181_-mediated sequence-specific RNA cleavage, Figure S2: Neutralization of MazF_NE1181_-mediated RNA cleavage, Table S1: Twenty-five sequences with MazF_NE1181_ cleavage, Table S2: Genes consisting of *mer* operon.

## Abbreviations

The following abbreviations are used in this manuscript:

TA	toxin-antitoxin
6-FAM	6-carboxyfluorescein
BHQ-1	black hole quencher-1
RASTA-Bacteria	rapid automated scan for toxins and antitoxins in bacteria

## Appendix A

The five artificially designed RNAs (1000-1, 1000-2, 1000-3, 1000-4, and 1000-5) including 1000-nt diverse sequences were fragmented with MazF_NE1181_. These RNAs were sequenced using the Illumina MiSeq platform with slightly modified RNA-seq protocols. The sequencing reads, which contained cleavage sites in their 5'-ends, were mapped against five reference sequences. Nucleotides showing increases in coverage were consistent with the first base of the cleaved RNAs (Figure 2A). These nucleotides were differentially detected by extracting the positions showing a large relative coverage increase, which is the value defined as the coverage at the n + 1^th^ position divided by the coverage at the n^th^ position. Sequences located five bases upstream and five bases downstream of these nucleotides were then extracted (Table S1) as outlined in our previous study [25]. A total of 25 sequences (five sequences derived from five references) were analyzed using WebLogo.

## Appendix B

Fluorogenic oligonucleotides modified with 6-carboxyfluorescein (6-FAM) at the 5′-end and black hole quencher-1 (BHQ-1) at the 3′-end were used in this study. The fluorescence from 6-FAM is typically quenched by BHQ-1, but when oligonucleotides are cleaved, these two dyes are no longer in close proximity and fluorescent intensity increases [25,34].
