# Supplementary material for: AAU-Specific RNA Cleavage Mediated by MazF Toxin Endoribonuclease Conserved in Nitrosomonas europaea
Source: Toxins (Basel). 2016 Jun 4;8(6):174. doi: 10.3390/toxins8060174 (PMC4926141; doi:10.3390/toxins8060174)
Supplement: Supplementary file 1 [file toxins-08-00174-s001.pdf]

Supplementary Materials: AAU-Specific RNA Cleavage Mediated by MazF Toxin Endoribonuclease Conserved in *Nitrosomonas europaea*

Tatsuki Miyamoto [1,2], Akiko Yokota [2], Satoshi Tsuneda [1,*] and Naohiro Noda [1,2,*]

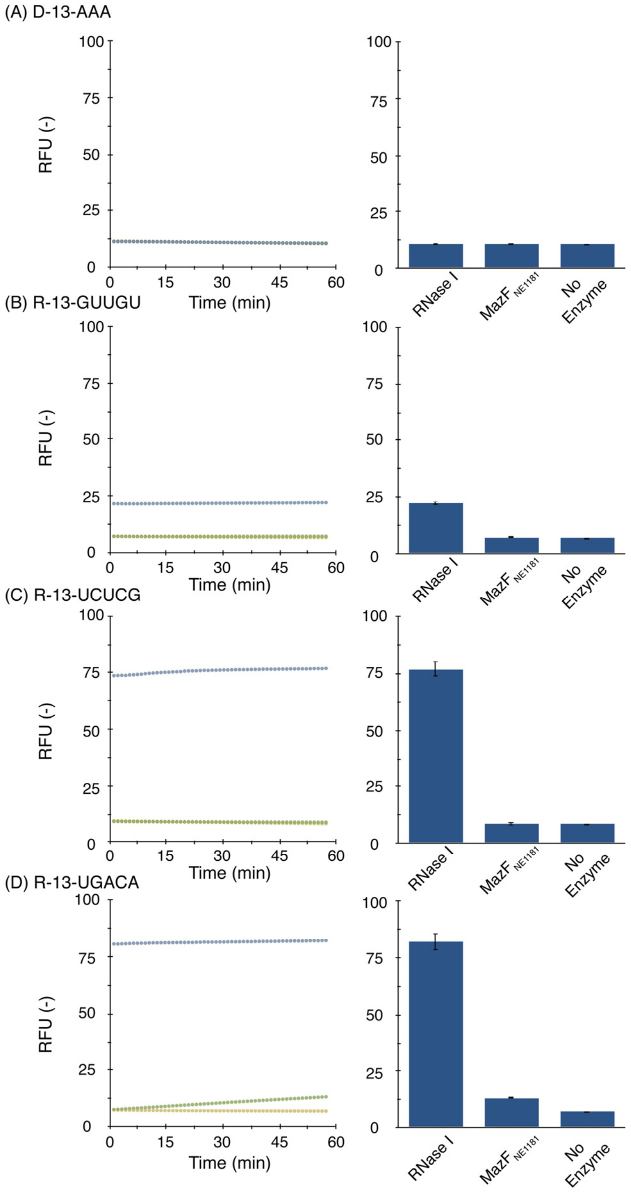

Figure S1. $MazF_{NE1181}$-mediated sequence-specific RNA cleavage. Two hundred nanograms of $MazF_{NE1181}$ (green) was incubated with 20 pmol of fluorescent-modified oligonucleotides; (**A**) D-13-AAA; (**B**) R-13-GUUGU; (**C**) R-13-UCUCG; and (**D**) R-13-UGACA. In the control reactions, fluorescent intensities in the presence of 1 U of RNase I (blue) and in the absence of enzymes (yellow) at each time point (left) and end point (right) were measured.

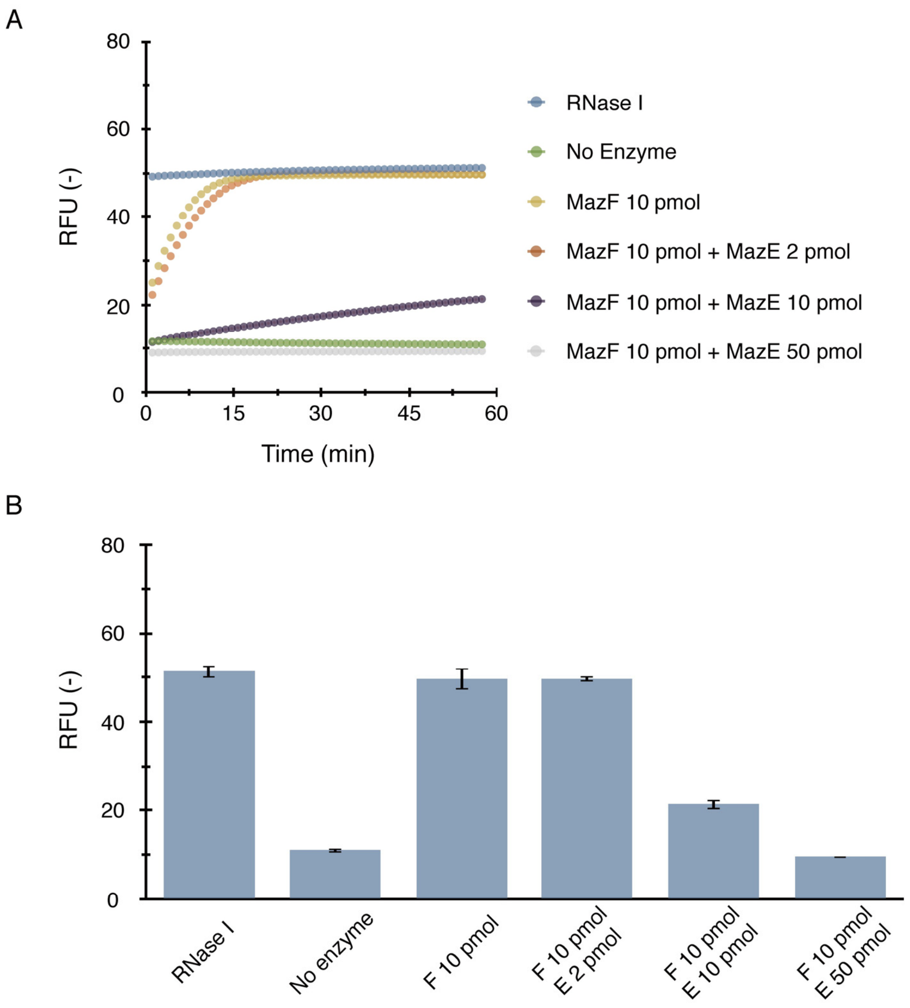

Figure S2. Neutralization of MazF$_{NE1181}$-mediated RNA cleavage. Ten picomoles of MazF$_{NE1181}$ were pre-incubated with 2 (vermillion), 10 (purple), or 50 (gray) pmol of MazE$_{NE1182}$, and mixed with 20 pmol of DR-13-AAU. In the control reactions, fluorescent intensities in the presence of 1 U of RNase I (blue) and MazF$_{NE1181}$ (yellow) and in the absence of enzymes (green) were measured. (**A**) Fluorescent intensities at each time point; (**B**) Fluorescent intensities at end point.

Table S1. Twenty-five sequences with MazF$_{NE1181}$ cleavage

RNA Type	Rank	Position	Relative Coverage Increase	Coverage	Sequence (5′ to 3′) [a]
1000-1	1	725	4.93	25,940	CCCAA<u>A</u>TAGAC
	2	332	2.53	14,909	CAGAA<u>A</u>TCACT
	3	188	2.11	5407	CCTAA<u>A</u>TGGAC
	4	392	1.78	22,871	CGCCA<u>A</u>TCTCT
	5	703	1.68	5667	CGAGA<u>A</u>TCATG
1000-2	1	71	5.12	1151	ACCGA<u>A</u>TCCCT
	2	192	2.49	3703	ATTTA<u>A</u>TGTTC
	3	461	1.79	6739	CTTCA<u>A</u>TTTGT
	4	277	1.75	5066	TCGTA<u>A</u>TGGTT
	5	628	1.68	16,116	GCGCA<u>A</u>AGGAC
1000-3	1	533	2.19	1472	AATGA<u>A</u>TATCG
	2	808	1.89	1061	TCCCA<u>A</u>TTCAA

	3	218	1.39	1067	TTGAAATCACC
	4	849	1.39	1294	TTCGAATTTCG
	5	726	1.36	1015	ATTCAATCTAC
	1	98	52.27	9200	TCCCAATAGTT
	2	480	2.88	10,727	CCTGAATACAC
1000-4	3	663	2.21	7650	CCTTAATAAGC
	4	335	2.06	4102	GCCCAATACGT
	5	798	1.81	3956	AGCTAATCGGA
	1	36	27.44	1427	TCGGAATCTTT
	2	715	9.34	8969	CATGAAATGAA
1000-5	3	432	1.81	18,470	CGCGAAAGGAT
	4	716	1.61	14,443	ATGAAATGAAC
	5	362	1.58	9653	TACGAATGGGC

[a]: Underlined letters represent the base with significant coverage increase.

Table S2. Genes consisting of *mer* operon.

Locus	Gene Symbol	Product Name	Length (bp)	Number of AAU Triplets
NE0842	*merT*	mercuric transport protein	351	0
NE0841	*merP*	mercury scavenger protein	276	0
NE0840	*merC*	mercury transport protein	423	2
NE0839	*merA*	mercuric reductase	1686	9
NE0838	*merD*	transcriptional regulator, MerR family	366	2
NE2575	*merE*	mercury resistance protein	237	0
NE0843	*merR*	transcriptional regulator, MerR family	426	4
